# Relationship between ovarian cancer stem cells, epithelial mesenchymal transition and tumour recurrence

**DOI:** 10.20517/cdr.2019.76

**Published:** 2019-12-19

**Authors:** Monica Angelica Amaya Padilla, Mudra Binju, Graeme Wan, Yohan Suryo Rahmanto, Pritinder Kaur, Yu Yu

**Affiliations:** ^1^School of Pharmacy & Biomedical Science, Curtin University, Curtin Health Innovative Research Institute, Perth, WA 6102, Australia.; ^2^Sidney Kimmel Comprehensive Cancer Center, Johns Hopkins Medical Institutions, Baltimore, MD 21231, USA.; ^3^Department of Pathology, Johns Hopkins Medical Institutions, Baltimore, MD 21205, USA.; ^4^Division of Obstetrics & Gynaecology, University of Western Australia Medical School, WA 6009, Australia.

**Keywords:** Ovarian cancer, stem cells, chemoresistance, platinum resistance, recurrence, epithelial-to-mesenchymal transition, extracellular vesicles

## Abstract

Investigating the biological processes that occur to enable recurrence and the development of chemoresistance in ovarian cancer is critical to the research and development of improved treatment options for patients. The lethality of ovarian cancer is largely attributed to the recurrence of disease with acquired chemoresistance. Cancer stem cells are likely to be critical in ovarian cancer progression, contributing to tumour malignancy, metastasis and recurrence by persisting in the body despite treatment with anti-cancer drugs. Moreover, cancer stem cells are capable of mediating epithelial-to-mesenchymal transition traits and secrete extracellular vesicles to acquire therapy resistance and disease dissemination. These attributes merit in depth research to provide insight into the biological role of ovarian cancer stem cells in disease progression and chemotherapy response, leading to the development of improved biomarkers and innovative therapeutic approaches.

## Introduction

The cancer stem cell (CSC) model has been proposed as a mechanism in which cancer cells gain the ability to persist despite the use of cytotoxic or targeted therapeutic approaches^[1]^. Although it is not yet clear how widely applicable this model is for different cancer types, the theory has helped to shape understanding of advanced cancers with high relapse rate after treatment. Applying the CSC theory in clinical translation is hampered by a lack of consensus on reliable CSC markers and their dynamic behaviour.

The focus of this article is ovarian cancer-stem like cells. We hypothesise that advanced epithelial ovarian cancer (OC) is a suitable model to explore CSC theory because of the high rate of tumour recurrence after conventional treatment. High-grade serous ovarian carcinoma is a major histological subtype of epithelial OC and the leading cause of burden among gynaecological cancers. Although patients usually have a positive response to first-line treatment, the cause for concern is the recurrence of disease that 70% of patients with advanced OC experience^[2]^.

The standard treatment option offered to OC patients involves chemotherapy with a platinum-based agent, although most patients eventually develop resistance to it once the disease recurs. Delving deeper into the mechanisms and the biological processes that enable recurrence and resistance is vital to the development of improved treatment options. Many hypothesise that the role of CSCs is critical to the recurrence of OC and could also encourage the development of chemoresistance^[1]^. Previously, we outlined a number of ovarian cancer stem cells (OCSCs) markers and their role in drug resistance^[3]^. In this review, we expand our discussion by exploring where the CSC model comes into play in OC recurrence, the metastatic process of OC and its crosstalk to promote cancer progression through mechanisms involving extracellular vesicles (EVs).

## The onset of ovarian cancer recurrence and cancer stem cells hypothesis

OC refers to the general anatomical location from which the cancer is derived; this can include the fallopian tubes, omentum, peritoneum and other surrounding tissues in addition to the ovaries. OC is typically diagnosed at advanced stages (III or IV), at which point the cancer has affected both ovaries and/or peritoneal metastasis has begun^[4]^. The dissemination of OC occurs through malignant ascites within the peritoneal cavity which lacks an anatomical barrier to obstruct the path of metastasis. The result is that solid tumour nodules spread and attach at secondary sites such as the omentum, intestines, spleen, bladder, etc. Despite being diagnosed at advanced stages of the disease, primary intervention with platinum-based therapy is initially effective in > 80% of epithelial OC patients^[5]^. Notably, standard treatment of OC with paclitaxel, cisplatin and carboplatin elicits a positive response in patients; however, only 10%-15% achieve full remission of the disease long-term^[6]^. It is for this reason that the lethality of OC is attributed to its recurrence with acquired chemoresistance in approximately 70% of advanced OC patients^[2,7]^. The recurrence of OC is characterised in terms of the patient’s platinum-free interval (PFI). The PFI defines the period of time elapsed between the completion of platinum-based chemotherapy to the detection of tumour relapse and importantly and is an accurate reflection of the patient’s anticipated response to second-line chemotherapy^[8]^. Subsequently, the PFI is utilized to determine the optimal second-line treatment regimen for the patient. Intervals are divided into four timeframes, namely “platinum refractory”, “platinum resistant”, “platinum sensitive” and “partially platinum sensitive”, where relapse occurs either within four weeks, less than six months, more than six months and within 6-12 months, respectively^[9,10]^. Patients who have developed chemoresistance to platinum-based agents have limited options, as the routinely used chemotherapeutic approaches would be ineffective. Further research into how OC cells acquire platinum resistance is essential when it comes to opening the door to improved treatment options for these patients.

The CSC hypothesis presents the idea that a subset of tumorigenic cells has the capacity to drive tumour progression and metastasis, consequently leading to relapse in patients^[11]^. Abubaker *et al*.^[12]^ administered *in vitro* chemotherapy to OVCA433 and HEY cell lines with cisplatin and paclitaxel. The results demonstrated an enhanced expression of CSC markers at both protein and mRNA levels post-chemotherapy^[12]^. Additionally, other researchers have corroborated the increased expression of CSC markers post-chemotherapy, reinforcing this finding^[12-14]^. In terms of relapse, it is important to evaluate the impact of CSCs because even patients who demonstrate a positive response to chemotherapy could relapse if a small population of CSCs were to persist^[15]^. Research has linked the presence of CSCs to chemoresistance and recurrence in OC based on the expression of stem cell markers such as CD24, CD44, CD117, CD133 and aldehyde dehydrogenase (ALDH)^[3,13,16-21]^. These findings reinforce the importance of CSC markers as biomarkers for the progression of OC [Table 1]. Collectively, these data suggest a relationship between the administration of platinum-based agents and the accumulation of stem cell characteristics, which in turn promote recurrence. However, further research must be done to better understand the efficacy of treatment and to ascertain the prognostic significance of using these markers. It is vital to evaluate the molecular mechanisms surrounding CSCs and their association with OC recurrence.

**Table 1:** The significance of OCSCs to recurrence and chemoresistance

Marker	Significance	Cell lines/Specimens	Ref.
CD24	Cytoplasmic localisation of CD24^+^ is indicative of poor prognosis and predictive of recurrence	Human ovarian serous tumours	[16]
	Increased metastasis and chemoresistance in CD24^+^ cells that have developed cisplatin-resistance	CAOV-3 cell line	[17]
CD44	CD44^+^/CD24^-^ expression in cells is predictive of recurrence	Human OC cells isolated from ascites	[18]
CD117	CD117^+^/c-Kit^+^ expression in cells is characteristic of chemoresistance	Human advanced serous ovarian carcinoma	[19]
	Therapy with paclitaxel increases CD177 expression *in vivo*	Primary tumour xenografts	[20]
CD133	Higher cisplatin IC_50_ in CD133^+^ cells characteristic of chemoresistance	42 established OC cell lines	[21]
ALDH	ALDH1A1 expression in OCSCs correlates with poor survival and chemoresistance	Human high-grade OC tumours	[13]

OC: ovarian cancer; OCSCs: ovarian cancer stem cells

## Epithelial-mesenchymal transition of ovarian cancer cells

**Figure 1:**
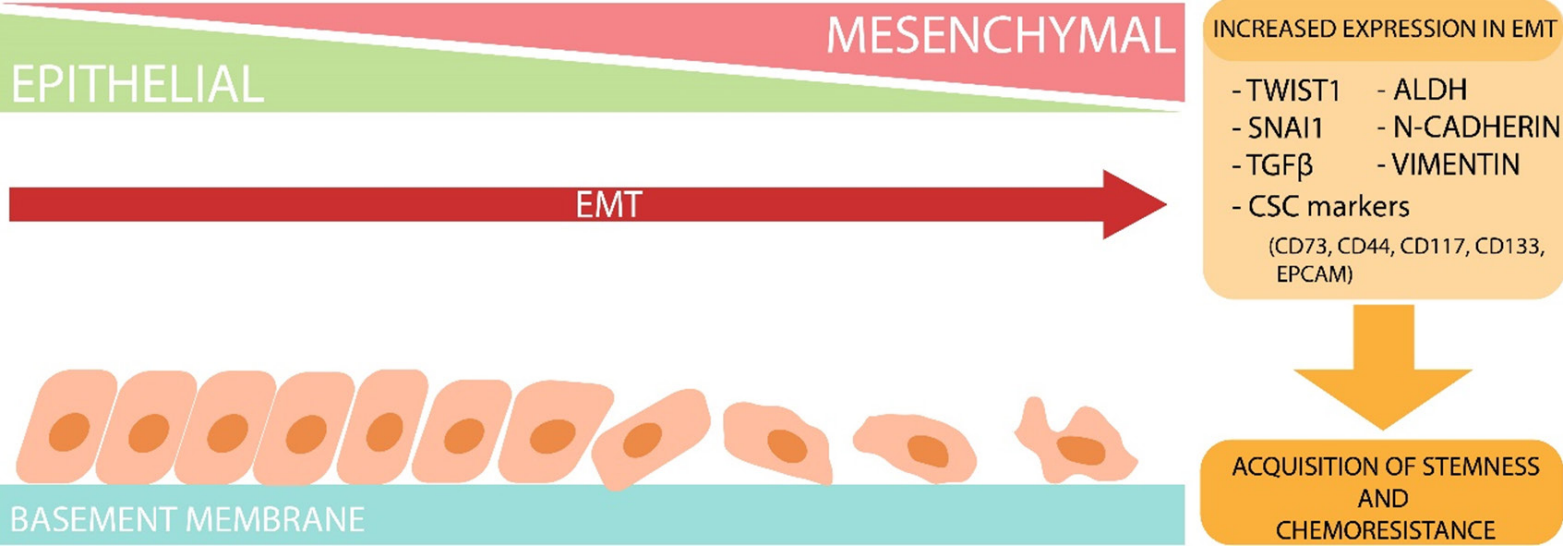
Epithelial-to-mesenchymal transition (EMT) process in cells. Diagrammatic summary depicting the effects of EMT in cells. The progression of EMT shows the upregulation of mesenchymal markers such as TWIST1, SNAI1, TGF-β, aldehyde dehydrogenase (ALDH), N-cadherin, vimentin and other cancer stem cell (CSC) markers. Profiles with increased expression of these markers have been shown to shift towards a mesenchymal phenotype depicted as irregular shaped cells with a lack of tight junctions. mesenchymal-to-epithelial transition is the reversion of this process. Cells that have shifted toward a mesenchymal state through the upregulation of these markers often acquire stem-like features and chemoresistance

Epithelial-to-mesenchymal transition (EMT) is a biological process in which epithelial cells have diminished cell-cell adhesion and cell polarity while they acquire invasive characteristics that promote a mesenchymal phenotype^[22]^. In embryogenesis, organ development and tissue regeneration EMT is an essential series of events that must occur in order to achieve normal development^[22,23]^. In terms of cancer, EMT is highly problematic as its acquisition enables cancer cell invasion and encouragement of metastasis^[24,25]^. Mesenchymal and epithelial characteristics exist on a spectrum whereby cells can present intermediate traits between the two extremes, rather than being completely epithelial- or mesenchymal-like cells^[26]^. The plasticity along the epithelial-mesenchymal axis permits the reverse process, which is termed mesenchymal-to-epithelial transition, shown in Figure 1^[27]^.

It is hypothesised that EMT is critical for OC progression. Immunohistochemical studies examining EMT-related proteins (E-cadherin and SNAI1) in primary and metastatic ovarian carcinoma showed that the EMT status, as marked by both a reduced E-cadherin expression and positive nuclear expression of *SNAI1*, was significantly higher in patients with peritoneal metastasis than those without. Notably, EMT-positive status was also a predictor of poor progression free survival and overall survival^[28]^. In addition, other EMT related proteins, such as vimentin and ZEB1, were identified as markers of poor chemotherapy response in metastatic serous ovarian carcinoma effusions^[29]^. Furthermore, a number of studies suggested that OC cells resistant to carboplatin and/or paclitaxel frequently acquire mesenchymal traits^[30-32]^. However, the relationship between OCSCs and acquisition of the mesenchymal phenotype is poorly understood.

## The idea of migrating cancer stem cells

The existence of stationary and migrating CSCs was initially suggested by Brabletz *et al*.^[33]^ to explain the behaviour of colon cancer. It is based on the observation that colon carcinoma gene expression can be largely grouped into a dynamic two-phase expression pattern: (1) stemness/proliferative group that is activated early and throughout the cancer progression; and (2) a EMT/dissemination group that is transiently activated later at the tumour-host interface^[33]^. Therefore, stationary CSCs are believed to be embedded in epithelial tissue, while mobile CSCs are predominantly at the tumour periphery at the host interface where they may readily undergo EMT to invade and metastasise, possibly through activation by environmental signals. The link between stemness and EMT is further supported by the findings that EMT activators such as TWIST1 can actually induce both EMT and “stemness” properties^[23]^.

Delving further into the associations between the existence of CSCs and EMT in OC, we find increasing evidence that supports the notion that OC cells with stem cell properties and/or EMT are inclined to develop resistance to therapy^[14]^. Moreover, the co-expression of “stemness” genes and EMT genes have been reported. Investigation of advanced-stage ovarian tumour sections, metastatic cells isolated from ascites and OC cell line revealed the combined expression of EMT and CSC markers after cisplatin treatment^[14]^. Cisplatin induced EMT morphology was characterised by reduced E-cadherin, as well as increased N-cadherin and vimentin expression. At the same time, CSC markers including CD44, CD117, CD133 and stem cell transcription factors Nanog and Oct-4 were also expressed. These studies utilised α2 integrin subunit and EpCAM to further characterise the cells on a molecular level^[14]^. A study by Lupia *et al*.^[34]^ using primary OC cells demonstrated the co-regulation of CD73, which was enriched in OC-initiating cells with EMT-associated factors such as SNAI1, TWIST1 and ZEB1 to promote a mesenchymal-like phenotype. CD73 causes dephosphorylation of adenosine monophosphate (AMP) to adenosine triggering an increase in intracellular cyclic AMP which is suppressive of immune functionality, consequently encouraging cancer progression, metastasis and angiogenesis^[35]^. Thus, CD73 promotes OC stemness and EMT^[34]^.

Research into the heterogeneity of OC cell lines opened the discussion about OCSCs establishing heterogeneity through EMT, which could result in metastasis, tumour progression or recurrence and drug resistance. Side population (SP) cells have tumour initiating capacity and express stem-like genes with characteristics that promote drug resistance^[36]^. Higher levels of SP cells have been detected in OC patients’ ascitic fluid, and these cells express mesenchymal gene expression patterns. The presence of these cells has been correlated with tumour grade which proves useful in a prognostic setting^[37]^. These observations implicate the EMT process in contributing to heterogeneity. Inhibiting the EMT process through *SNAI1* silencing reduced SP cells frequency, suggesting that *SNAI1*-regulated EMT may be required to maintain OCSCs^[36]^.

The processes of glycolysis and lipolysis are pivotal to the supply of energy to CSCs^[38]^. There is limited research into the relationship between EMT and these processes, however, we have highlighted some findings of interest in this review. The inhibition of glycolysis in cancer stem-like cells enriched from the SKOV3 cell line (i.e., treatment with Lonidamine) impeded the expression of EMT-associated genes (*ZEB, SNAIL* and *vimentin*)^[39]^. In contrast, only moderate effect was observed when perturbing lipolysis. Thus, inhibiting glycolysis under the right conditions could be used as a CSC target to prevent EMT.

A study by Marchini and colleagues compared the molecular profiles of stage III-IV epithelial ovarian tumours before and after several lines of platinum chemotherapy. They identified resistant gene expression signatures indicative of EMT activation via the TGFβ pathway^[38]^. Importantly, this gene signature also negatively affected survival in OC patients^[38]^. In cell line studies, the exposure of OC cells to prolonged treatment with TGFβ1 (48 h) resulted in the acquisition of stem-like characteristics and chemoresistant properties. TGFβ1 induced the mRNA transcription of pluripotent markers such as SOX2, OCT4a, NANOG, CD44 and CD117^[40]^. Furthermore, TGFβ1 treatment can upregulate the expression of *ABCG2*, a member of the ABC transporter family, at both the mRNA and protein level consistent with previous findings^[41]^. Upregulation of drug transporter proteins from the ABC family can be a hallmark of acquired chemoresistance given their well-documented drug efflux functions^[42]^. A recent report using cytotoxicity as a readout showed that human ovarian teratocarcinoma PA1 cells were susceptible to cisplatin induced cell necrosis - but not when treated with TGFb1. This suggests that the TGFb pathway ultimately affects the chemosensitivity of OC cells^[41]^. Interestingly, the use of a calcium channel blocker such as verapamil can re-sensitise TGFβ1-treated cells to the cytotoxic effects of cisplatin^[41]^. Analyses of the SP fraction of the HO-8910PM human epithelial OV cell line following TGFβ1 treatment revealed transformation to mesenchymal features, stimulating expression levels of the EMT-related genes *SNAI1, SNAI2* and *CDH2*^[43]^. These data propose the concept of OCSC evolution via EMT, which could consequently affect chemoresistance and disease progression.

OCSCs often demonstrate high expression of NANOG which influences EMT processing^[44]^. NANOG utilises the STAT3 pathway to encourage EMT and drug resistance, hence increasing its expression within chemoresistant OC cell lines^[45,46]^. It is even postulated that NANOG expression may be an explanation for the aggressive phenotype associated with OCSCs^[46]^. A putative drug resistant tumour cell phenotype, EpCAM^+^CD45^+^, was isolated from the ascitic fluid of OC patients. When compared to only EpCAM^+^ cells, the drug resistant phenotype demonstrates an increase in *E-cadherin, N-cadherin* and *vimentin* gene expression, which suggest the promotion of EMT and an association with chemoresistance^[47]^. EpCAM^+^CD45^+^ cells have a highly invasive phenotype with mesenchymal gene signatures largely attributed to an OCSC subpopulation. This further points to the impact of EMT in CSCs and how that causes drug resistance.

ALDH is another OCSC marker where expression is linked to poor prognoses and chemoresistance in OC patients^[13]^. ALDH^high^ OC populations demonstrate CSC-like invasive properties and express CSC markers *in vivo* while simultaneously demonstrating advanced stages of EMT processing^[48]^. Collectively, these findings support and give an insight into the complex interplay among EMT, CSCs and the onset of OC chemoresistance. The underlying hypothesis behind this is that, during therapy, OC tumour cells can acquire features that are characteristic of EMT and CSC signatures that assist and promote tumour recurrence [Figure 1].

## Extracellular vesicles

Interest in EVs and their role in intercellular communication in a cancer setting has gained traction in recent years. EVs can promote tumour cell growth, proliferation and metastasis in cancer, which has made them an attractive prospect in research^[49,50]^. EV is a blanket term that is used to describe any secreted vesicle including microvesicles, oncosomes, exosomes and many more. EVs are secreted by many cell types and can be detected in blood and urine. The contents of EVs vary depending on the cell that secretes them. They can contain proteins, nucleic acids and other biological material^[49]^. The biological material enclosed in the EV is transported in the bloodstream allowing cell-cell communication via these secreted vesicles.

**Figure 2:**
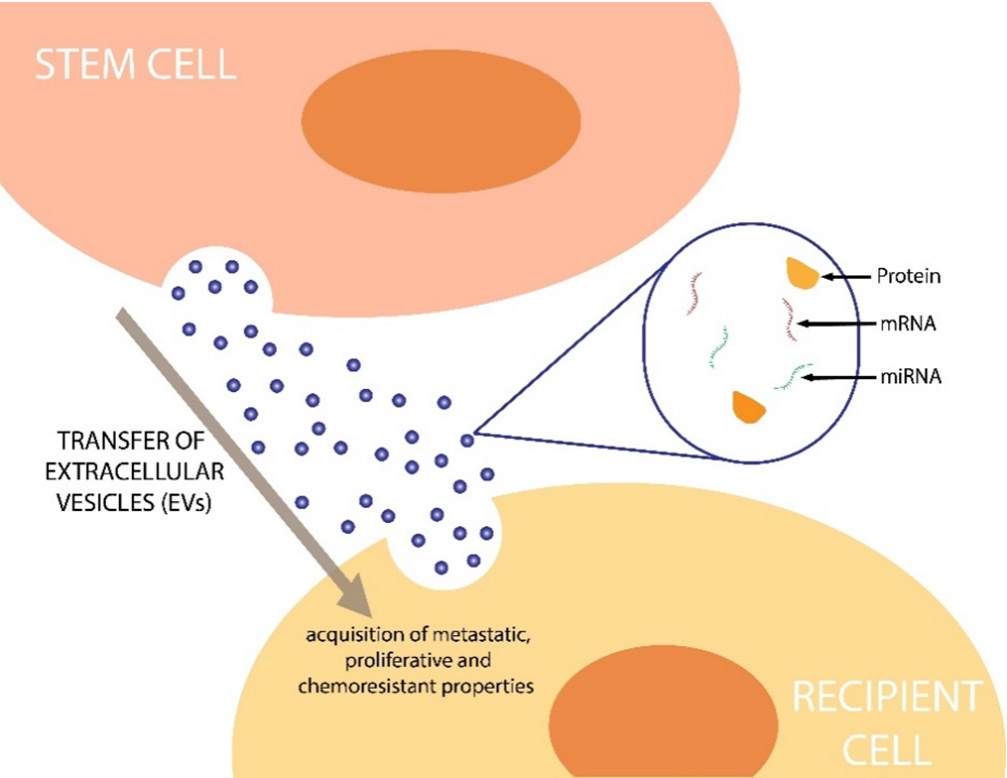
Transfer of extracellular vesicles (EVs) to recipient cells. A proposed model of the process that stem cells use to transfer EVs containing biological materials such as mRNAs, miRNAs and proteins, which can ultimately confer chemoresistant and cancer stem cell properties to recipient cells

There have been developments in the research of cancer-associated EVs that suggest they assist the transmission of mRNAs, miRNAs and proteins, conferring chemoresistance from cell to cell in many cancers including OC [Figure 2]. An early study by Safaei and colleagues utilised the lysosomal compartment in cisplatin-sensitive 2008 human ovarian carcinoma cells to demonstrate that EVs confer chemoresistance in a cisplatin-resistant subvariant cell line^[51]^. In this study, the 2008/C13*5.25 cisplatin-resistant variant exported 2.6-fold more cisplatin than the drug sensitive 2008 human ovarian carcinoma parental cell line^[51]^. When A2780 cells were treated with exosomes from the 2008/C13*5.25 cell line, they produced gene signatures coinciding with those of EMT. Crow and colleagues suggested that this may be achieved through the upregulation of EMT markers via secreted exosomes and EVs as a whole^[52]^.

Pluripotent stem cells have been shown to secrete EVs. For example, mouse cardiac fibroblast-derived induced pluripotent stem cells were reported to secrete cardioprotective miRNAs including NANOG-regulated miR-21 and HIF-1α-regulated miR-210 to prevent cardiomyocyte apoptosis^[53]^. Similarly, CSCs may exploit EVs to promote the progression of cancer by affecting cell-cell communication^[54]^. Cha and colleagues^[55]^ investigated miRNA dysregulation in CSCs isolated from human ovarian carcinoma tissues. CSCs derived from spheroid-forming primary cancer cells showed dysregulation of five miRNAs (miR-5703, miR-630, miR-1246, miR-424-5p and miR-320b) when compared to primary cancer cell expression profiles. In particular, miR-424-5p was linked to distant metastasis, suggesting a role in dissemination of disease^[55]^. Given that miRNAs control CSC self-renewal and differentiation, these findings suggest that miR-424-5p is a potential biomarker for poor prognosis in OC, making this an attractive target in terms of therapeutics.

A recent study by Srivastava *et al*.^[56]^ suggested that miRNAs are important in maintaining CSC properties. Specifically, OCSCs exhibited upregulated miR-328-3p (miR-328) through targeting DNA damage binding protein 2. Enhanced expression of miR-328 was related to reduced ERK signalling activity in OCSC, and miR-328 inhibition prevented tumour metastasis and growth in murine ovarian xenografts^[56]^. These findings suggest the possibility of targeting miR-328 to eradicate CSC in OC. In cell line studies, decreased expression of miR-34c-5p in SKOV-I6 and OVS1 cells resulted in docetaxel and carboplatin drug resistance^[57]^. Conversely, chemoresistant cell lines SKOV3/DDP and SKOV3 showed increased expression of miRNA-30a-5p compared to chemosensitive cell lines, reinforcing their role in chemoresistance^[58]^. These data demonstrate how regulatory miRNAs can be exploited to confer resistance and promote the progression of disease.

## Conclusion

Studies have demonstrated the enrichment of CSC markers following treatment with platinum-based agents. Treatment with platinum-based agents encourages the development of CSC characteristics which promote tumour progression and metastasis, ultimately resulting in wide-spread disease recurrence unamenable to secondary surgery. We hypothesise that treatment with chemotherapeutic agents can lead to alterations in molecular signatures typical of CSCs. As outlined, there is ample evidence that supports a relationship among chemotherapy, CSCs and resistance. The issue, however, is being able to determine the extent to which tumours recur because of chemotherapy, which induces CSCs to dominate, or due to CSCs’ intrinsic resistance to chemotherapy in OC. Migrating CSCs have the capacity to undergo EMT processing, negatively impacting patient disease progression. Furthermore, CSCs may promote cancer progression through stem cell associated EVs. At the same time, these cells are maintained by an EV-based mechanism that is crucial in governing cell-cell communication. It is vital that future studies investigate the interplay between CSCs and EMT in greater depth in order to understand metastatic tumour recurrence in advanced OCs.

## Declarations

### Copyright

© The Author(s) 2019.
